# Environmental Compatibility of *Penicillium rubens* Strain 212: Impact on Indigenous Soil Fungal Community Dynamics

**DOI:** 10.3390/jof11120852

**Published:** 2025-11-29

**Authors:** Belén Guijarro, Gema Vázquez, Antonieta De Cal, Paloma Melgarejo, Núria Gaju, Maira Martínez-Alonso, Inmaculada Larena

**Affiliations:** 1 Grupo de Hongos Fitopatógenos, Departamento de Protección Vegetal, Centro Nacional INIA-CSIC, 28040 Madrid, Spain; guijarro.belen@inia.csic.es (B.G.);; 2 Departament de Genètica i Mcrobiologia, Universitat Autònoma de Barcelona, 08193 Bellaterra, Barcelona, Spain

**Keywords:** *Penicillium rubens*, PO212, biological control agent, *Fusarium oxysporum*, environmental risk assessment, soil fungal community

## Abstract

Fusarium wilt causes substantial losses in many crops, and *Penicillium rubens* strain 212 (PO212) is a well-established biological control agent effective against several soil-borne pathogens, including the causal agents of Fusarium wilt. Before its widespread use, it is essential to assess whether applying PO212 may affect indigenous soil microbial communities. To address this, two open-field tomato trials were conducted to evaluate spatial and temporal changes in non-target soil fungal communities following the application of PO212. Fungal community profiles were monitored over one year using PCR–DGGE of fungal rDNA, and representative DGGE bands were sequenced for taxonomic confirmation. Community structure and variability were analysed using cluster analysis (UPGMA and Neighbor-Joining) and analysis of molecular variance (AMOVA) to determine the effects of treatment, sampling date, and soil depth. PO212 application did not significantly altered the composition or diversity of indigenous soil fungal communities. DGGE banding patterns and diversity indices were similar between treated and untreated soils throughout the study period. Observed community changes were driven primarily by temporal (seasonal) variation, with samples collected at 365 days clustering separately from earlier sampling dates for both treatments. AMOVA confirmed that sampling date, rather than PO212 treatment, explained most of the variance in community composition (*p* < 0.05). Although PO212 persisted in soil, fluctuations in other fungal populations were minor and within the range of natural seasonal variability. Overall, field application of PO212 did not disrupt indigenous soil fungal communities, supporting its environmental safety as a biocontrol agent for managing Fusarium wilt.

## 1. Introduction

Soil microorganisms—primarily bacteria and fungi—dominate soil biota in terms of biomass and diversity and play an important role in ecosystem functioning. They drive organic matter decomposition, nutrient cycling and fixation, influence plant productivity and health, improve soil structure, degrade pollutants, and regulate greenhouse gas emissions [1,2]. Given these crucial services, any disturbance in soil microbial communities can impair soil fertility by reducing nutrient availability, altering decomposition processes, and weakening the biological functions that support healthy plant growth, for example, disrupting decomposer fungi and bacteria slows organic matter breakdown, reducing nutrient release and limiting crop nutrient availability. Introducing a non-native organism such as a microbial biocontrol agent (BCA) into the soil could disrupt indigenous microbial populations; however, this risk has rarely been studied. Before implementing BCAs on a large scale, its environmental safety and effects on non-target soil microorganisms need to be assessed [3].

Direct or indirect impacts of an introduced BCA could include competition with indigenous microorganisms, production of antagonistic metabolites, or even parasitism of non-target species that are beneficial to plants. The soil microbial balance is delicate and disturbing it might have unintended consequences for plant health—for example, by reducing beneficial symbionts or disrupting microbial food webs. Such changes could be mediated not only by the introduced organism’s viable cells but also by its secondary metabolites in the soil [4]. Indeed, BCAs employ multiple mechanisms of action to suppress pathogens, including mycoparasitism, antibiotics and lytic enzymes production, competition for nutrients and niche space, induction of plant defenses, etc. While these mechanisms make BCAs effective against target pathogens, they could pose risks to closely related non-target microbes. For instance, *Trichoderma* spp., a common fungal BCA, are potent mycoparasites of fungi; this can include parasitism of beneficial fungi such as arbuscular mycorrhizal (AM) fungi. Studies Postman et al. [5] have shown that the introduction of a biocontrol *Trichoderma atroviride* strain into soil reduced AM fungal colonization of plant roots and altered native microbial populations. *Trichoderma* spp. can produce chitinases and other enzymes that degrade fungal cell walls, penetrate AM fungal spores and hyphae, and interfere with AM symbiosis [6]. This antagonism may harm the host plant by diminishing mycorrhizal benefits [7].

Fungal BCAs such as *Pythium oligandrum*, *Talaromyces flavus*, *Coniothyrium minitans*, and *Ampelomyces quisqualis* have also demonstrated modes of action (e.g., mycoparasitism, antibiotic production) that could potentially affect non-target fungi or bacteria in soil [8]. Some studies have also shown that fungal BCAs can have negative effects on beneficial rhizosphere bacteria. For example, inoculation with certain saprophytic fungi inhibited nitrogen-fixing and *Rhizobium* nodulation in legumes [9,10]. *Bacillus thuringiensis*, a bacterial BCA mainly targets insects, has also been reported to significantly alter the structure of the microbial community in the phyllosphere when applied to plant foliage [11].

In general, the most frequently reported non-target effect of BCAs is a temporary reduction in the abundance or diversity of indigenous soil microbiota immediately after application. This effect is usually short-lived and diminishes as the introduced agent declines or adapts in the soil [12]. Environmental factors such as soil type, moisture, and temperature, as well as the resilience of native communities, often influence these outcomes. These factors can facilitate recovery to pre-treatment community states [13].

*Penicillium rubens* (historically referred to as *P. chrysogenum*) and *P. oxalicum* are naturally occurring, cosmopolitan fungi that are frequently isolated from diverse soils including agricultural, forest, and rhizosphere environments. Numerous surveys have reported the autochthonous presence of *Penicillium* spp. in soil, with background concentrations ranging from 10^2^ to 10^3^ colony-forming units (CFU) per gram of soil under untreated conditions [14]. Molecular studies also confirm their frequent detection as part of the general *Penicillium* spp. assemblage [15,16]. This widespread distribution suggests that these fungi are integral components of many soil microbial communities, participating in nutrient cycling and organic matter decomposition.

*P. rubens* strain 212 (PO212) (ATCC 201888), formerly *P. oxalicum*, is an example of a promising fungal BCA originally isolated from Spanish soils. Previous studies demonstrated that PO212 effectively controls a broad range of economically important soil-borne pathogens for horticultural crops in nurseries, greenhouses, and open fields [17,18,19] underlining its broad biocontrol spectrum Notably, PO212 suppresses tomato vascular wilt caused by *Fusarium oxysporum* f. sp. *lycopersici*. This strain is a saprophytic and cosmopolitan fungus with a highly competitive fitness in the soil, tolerating a wide range of pH and moisture conditions [15,20,21,22]. These traits contribute to its success as a BCA across different environments. The biocontrol agent induces the plant’s defences against Fusarium wilt in tomato, thereby increasing production. It should be noted that these pathogens reduce the productivity of the crops they affect. Therefore, PO212 has also been used as a plant growth promoter. PO212 was reclassified as a strain of the specie *P. rubens* by Villarino et al. [23].

Overall, while microbial BCAs are generally considered as low-risk pest control options, their potential effects on soil ecosystem functions should be carefully considered [15]. Under the current EU regulatory framework, specific assessments of impacts on non-target soil microorganisms are not explicitly required for approval; however, such effects may become relevant in the context of ecosystem services evaluation [24]. In this context, the present study examines the impact of PO212 applications on the structure of fungal soil communities in tomato crops. We hypothesized that PO212, as a primarily saprophytic fungus, would integrate into the soil with minimal disturbance to native fungal assemblages.

Among the molecular tools available for monitoring such changes, Polymerase Chain Reaction–Denaturing Gradient Gel Electrophoresis (PCR-DGGE) remains a valuable and well-suited method for rapidly comparing microbial communities subjected to different treatments through the analysis of many of samples. PCR-DGGE provides a fingerprinting approach that highlights changes in dominant microbial populations in response to biotic or abiotic disturbances [25,26], allowing efficient comparison for ecological impact assessment. DGGE focuses on ecologically meaningful variation and thus offers an appropriate and practical method for screening fungal community responses to the introduced organism. Similar approaches have been used to study non-target effects of other BCAs, such as *Trichoderma* and beneficial rhizobacteria, on soil microbial community diversity [27]. By employing this DNA fingerprinting technique, we aim to determine whether PO212 causes any measurable shifts in fungal community structure or whether any observed changes are instead attributable to natural spatial-temporal variability. Our findings will help inform environmental risk assessments and support the safe registration and use of PO212 as a biocontrol product.

The objective of this study was to evaluate the potential effects of the fungus *Penicillium rubens* strain 212 on the structure and diversity of soil fungal communities under field conditions. Soil health is a cornerstone of sustainable agriculture. Any disturbance to its microbial networks can have a cascading effect on nutrient cycling, plant growth, and long-term productivity as well as altering other soil ecosystems. Therefore, it is essential to determine whether the application of PO212 may lead to unintended ecological impacts, such as reduced microbial diversity or disruption of beneficial fungal populations. By combining field experimentation with molecular diagnostics, we aim to generate data to guide both the practical implementation of biocontrol and environmental risk assessments for microbial products.

## 2. Materials and Methods

### 2.1. Culture Conditions and Plant Material

PO212 was maintained on potato dextrose agar (PDA; Difco, Detroit, MI, USA) slants at 4 °C (short-term) and in 20% glycerol stocks at −80 °C (long-term). For inoculum production, PO212 was grown on PDA plates at 20–25 °C in the dark for 7 days to induce abundant conidiation. Mass production of PO212 conidia was achieved by solid-state fermentation on a lentil/vermiculite substrate, following the method of De Cal et al. (2000) [18]. This method consistently yields ~10^8^ conidia per gram of dry substrate with 80–90% conidial viability. After incubation, fresh conidia were harvested and dried in a fluidised bed dryer (Burkard 350S, Burkard Manufacturing Company Limited, Hertfordshire, UK) to <10% moisture for formulating as a dry powder inoculant (referred to as CSPO).

Tomato (*Solanum lycopersicum*) cv. San Pedro (susceptible to *F. oxysporum f.* sp. *lycopersici* races 1 and 2) was used for the field trials. Tomato seedlings were raised in trays with a vermiculite–peat mixture in a growth chamber (22–28 °C, 16 h photoperiod, high humidity) for 3–4 weeks. One week before transplanting to the field, seedlings designated for the biocontrol treatment were inoculated with PO212. CSPO was suspended in 200 mL of sterile distilled water for each seedbed, containing 40 seeds. This aqueous solution was applied at a final concentration of 1 × 10^7^ conidia/g of substrate [28,29,30]. Control treatments were treated with sterile distilled water.

### 2.2. Field Trials and Sampling

Two field trials were conducted in the Madrid region of Spain during tomato growing seasons (May–November). Each trial was established as a randomized complete block design with four replicate plots per treatment (PO212-treated vs. untreated control). Each replicate plot (24 m^2^ area) contained 40 tomato plants, spaced 1 m × 1.2 m apart in rows. One trial site was in a commercial orchard in Villaviciosa de Odón, southern Madrid, Spain (Coordinates: 40°21′17″ N, 3°53′55″ W), hereafter referred to as VO. The soil at this site is a natural sandy loam with a pH of approximately 6.3. The second site, referred to as LC, was located at the La Canaleja farm in Alcalá de Henares, eastern Madrid, Spain (Coordinates: 40°28′53″ N, 3°21′50″ W). This site features loamy sand soil with a pH of approximately 8.5. The key physico-chemical properties of the two soils are described in Table S1. The mean annual temperatures for the VO and LC fields was 13.2 °C and 13.7 °C, respectively, and the mean annual precipitation in both fields ranged between 400 and 470 mm. Prior to transplanting, both fields were fertilised with 40 t/ha of well-aged horse manure (containing approximately 1.2% nitrogen (N), 0.6% phosphorus (P_2_O_5_), 1.5% potassium (K_2_O) and 45% organic matter), which was applied three months before planting, and prepared using standard tillage methods. No fungicides were applied in either trial for the duration of the study. Plants were drip-irrigated as needed. To control pests, a single application of insecticides/acaricides (chlorpyrifos, dicofol, and abamectin) two months after planting was made at VO, and a single application of *Bacillus thuringiensis* (kurstaki strain) one month after planting was made at LC. Weeds were managed by mechanical cultivation in all plots.

Tomato seedlings were transplanted into field plots in early summer. In the treatment plots, each seedling had been pre-inoculated with PO212 as described above. The application rate used corresponds to the effective agronomic dose required for induction of resistance and is the only rate relevant for environmental risk assessment [18,19,20,21]. Control plots were planted with identical but untreated seedlings. To assess the impact of PO212 on soil fungal communities over time, soil sampling was performed at 0, 75, 180, and 365 days after transplanting (i.e., at planting, mid-season, end of season, and one year later). At each sampling time, soil cores were collected from both the surface layer (0–5 cm depth, denoted “S”) and the subsurface root zone (6–10 cm depth, denoted “P”) to analyse microbial variability between the biologically active surface layer and the more stable, root-influenced subsurface zone in each plot. Within each plot, samples were taken at three randomly selected points around different tomato plants. Each sample was a composite of five soil cores (20 mm diameter) taken within 30 cm of the base of a tomato plant (to target the rhizosphere vicinity). The five cores from each depth and plot were pooled and thoroughly homogenised to form one composite sample. This process was repeated for three plants per treatment per time point, yielding three independent composite samples for each depth × treatment × time combination. Samples were placed on ice for transport to the lab, then stored at −20 °C prior to DNA extraction.

### 2.3. DNA Extraction and PCR Amplification

Total community DNA was extracted from 10 g of each soil composite sample using the FastDNA™ SPIN Kit for Soil (MP Biomedicals, Solon, OH, USA) following the manufacturer’s protocol, with an added bead-beating step to improve cell lysis. Briefly, soil aliquots were subjected to three rounds of bead-beating (FastPrep 24 instrument, MP Biomedicals) at 5.5 m/s for 60 s each. This rigorous mechanical lysis step enhanced DNA yields, especially from fungal spores and hyphae. The extracted DNA was checked on agarose gels for quality and stored at −20 °C.

Fragments of 18S rRNA gene (covering the ITS regions) from the fungal community were amplified by using a nested PCR. In the first-round PCR, the universal fungal primer set EF4 (forward, 5′-GGAAGGGRTGTATTTATTAG-3′) [31] and ITS4 (reverse, 5′-TCCTCCGCTTATTGATATGC-3′) [32] was used to amplify ~999 bp of the 18S–ITS region. The second-round PCR was then performed with the primer pair targeting an internal 300 bp ITS region, ITS1F-GC (forward, 5′-TTGGTCATTTAGAGGAAGTAA-3′) [33] with a 40 bp GC-rich clamp added to its 5′ end that prevents complete strand dissociation during DGGE and ITS2R (reverse, 5′ GCTGCGTTCTTCATCGATGC 3′) [32].Reaction mixtures (50 µL) contained 10 mM Tris–HCl (pH 8.3), 50 mM KCl, 2.5 mM MgCl_2_, 0.25 mM each dNTP, 2 µL of BSA (20 µg/µL), 25 pmol of each primer, 2.5 U of Taq DNA polymerase (Biotools B&M Labs, Madrid, Spain), and ~5–20 ng of template DNA. PCR cycling conditions for both rounds were: 94 °C for 5 min; 35 cycles of 94 °C for 30 s, 55 °C for 30 s, 72 °C for 30 s; and a final 5 min extension at 72 °C [26]. Positive (template DNA from pure PO212 culture) and negative controls (no template) were always included to verify reaction efficacy and rule out contamination, respectively. All PCR reactions were carried out in a Biometra T-Personal 48 thermal cycler. Finally, PCR products were analyzed by electrophoresis on 1.5% agarose gels in TBE buffer (Tris borate EDTA: TRIS base, 89 mM; boric acid, 89 mM; and EDTA, 2 mM), stained with 0.5 ppm of ethidium bromide, and visualized under UV light to confirm successful amplification. Consistent bands of the expected size were obtained from all samples and quantified using Quantity One 1-D analysis software version 4.6.9. The Low DNA Mass Ladder from Invitrogen™ (Carlsbad, CA, USA) was used as a standard for quantification on agarose gels.

### 2.4. DGGE Profiling and Sequence Analysis

DGGE analysis was performed using a DCode™ Universal Mutation Detection System (Bio-Rad Laboratories, Hercules, CA, USA). For each sample, a total of 1200 ng of each PCR product was loaded on 6% (*w*/*v*) polyacrylamide gels (37.5:1 acrylamide-N:N′-methylene-bis-acrylamide) with a denaturing gradient of 20% to 60% urea–formamide (where 100% denaturant contains 7 M urea and 40% deionized formamide). Positive control (template PCR-DNA from PO212 culture) was loaded in both DGGE gel, allowing the direct comparison of the fingerprints. This enabled us to observe its presence over time by the band position. Electrophoresis was carried out in TAE 1X buffer (40 mM Tris-acetate [pH 7.4], 20 mM sodium acetate, and 1 mM EDTA) for 12 h at 60 °C and 75 V. After electrophoresis, gels were stained in ethidium bromide (0.5 µg/mL), rinsed, and photographed in a Bio-Rad Gel Doc system.

To identify the most prominent fungal members inthe community, the clearly defined DGGE bands were recovered from the gels and re-amplified using the primers ITS1F (without GC clamp) and ITS2R under the same conditions as before. The re-amplified products were purified (using a PCR cleanup kit, UltraCleanTM PCR cleanup DNA, MoBio laboratories Ink, Carlsbad, CA, USA) and Sanger-sequenced by a commercial service (San Sebastian de los Reyes, Madrid, Spain). The resulting sequences were trimmed and quality-checked using BioEdit 5.0.9, aligned using ClustalX 2.1, and verified with BLAST 2.17.0 alignment against databases, with a similarity threshold of >92% [34]. A >92% similarity threshold was considered given the good quality of the sequences obtained. Curated sequences were deposited in the National Center for Biotechnology Information (NCBI) GenBank database under submission number SUB15759917.

### 2.5. Data Analysis

Digital images of DGGE gels were analysed to compare community profiles across treatments, depths, and times. Each distinct band position on the gel was treated as an operational taxonomic unit (OTU). For all genetic profiles the total number of bands were determined, and a distance matrix was constructed using the Dice coefficient (also known as Sørensen’s similarity), which emphasises shared bands between two samples. These similarity/distance measures provided the basis for multivariate analyses of community structure [35].

To visualise relationships among samples, hierarchical cluster analyses were performed using two clustering methods: (a) the unweighted pair-group method with arithmetic mean (UPGMA) and (b) the Neighbor-Joining (NJ, a minimum-evolution tree method). UPGMA clustering of the Dice similarity matrix was performed with the NTSYSpc software (v2.10b). The cophenetic correlation (Mantel test) was calculated to assess how well the dendrogram represented the similarity data. NJ clustering was performed on the Nei’s distance matrix using GenAlEx (v6.2). As both methods produce very similar sample groups, only the results of one method are shown.

We used Analysis of Molecular Variance (AMOVA) to quantitatively partition the observed variance in community composition among different factors. AMOVA [36,37] was run in GenAlEx v6.2 [38] to test for significant differences between (i) treatment groups (PO212-treated vs. control), (ii) soil depths (surface vs. subsurface), and (iii) sampling times (0, 75, 180, 365 d). The binary community matrix was used to calculate variance components among and within groups, and an FST-like statistic was obtained to measure the degree of differentiation. Significance of variance components was assessed by permutation tests in GenAlEx (using 999 permutations). Additionally, principal component analysis (PCA) on the community data was performed to explore patterns without a priori grouping. PCA was done with StatGraphic Centurion XVI.V16.103 (Stat Point, Ink Warrigton, WA, USA) using the prcomp function on the presence–absence data (treating each band as a variable). Environmental data (soil temperature, moisture, sampling date and depth) and presence of PO212 were included as supplementary variables in the PCA to infer correlations with community shifts.

Ambient temperature ($T_{\mathrm{amb}}$) and relative humidity data were provided by AEMET (Spanish State Meteorological Agency, Ministry of Agriculture, Food and Environment). Soil plough layer temperature was calculated from ambient temperature using the following equation, taken from the Technical Guide: Outdoor Climatic Conditions for Design (https://transparencia.gob.es/en/publicidad-activa/por-materias/organizacion-empleo/normativa/normativa-mitu (accessed on 20 November 2025)): where $T_{\mathrm{amb}}$ is the daily mean ambient temperature and $T_{\mathrm{soil}}$ is the resulting soil temperature for each day.

$$T_{\mathrm{soil}}=0.0068\;T_{\mathrm{amb}}^{2}+0.963\;T_{\mathrm{amb}}+0.6865$$

## 3. Results

### 3.1. Fungal Community Profiles and Diversity

Across all soil samples, DGGE profiling revealed a complex fungal community, with numerous bands per sample. In the initial soil samples (time 0), both control and PO212-amended plots displayed rich band patterns (Figure S1a,b), indicating diverse baseline fungal communities. In total, 413 and 168 DGGE bands were detected across samples in the VO and LC samples, respectively, typically around 23–25 distinct band positions per gel in each trial. A total of 56 DGGE bands were excised and sequenced from the VO samples, and 46 bands from LC, representing the dominant fungal phylotypes in those communities (Figure S1a,b).

PO212 treatment did not significantly alter fungal community richness or evenness. Both control and treated soils exhibited similar numbers of bands and comparable band intensity patterns at all sampling points. Differences observed between treatments were minimal compared to the natural variation across sampling times and soil depths. Likewise, overall community diversity, as reflected by the DGGE profiles, remained consistent regardless of PO212 application (Figure S1a,b).

**Figure 1:**
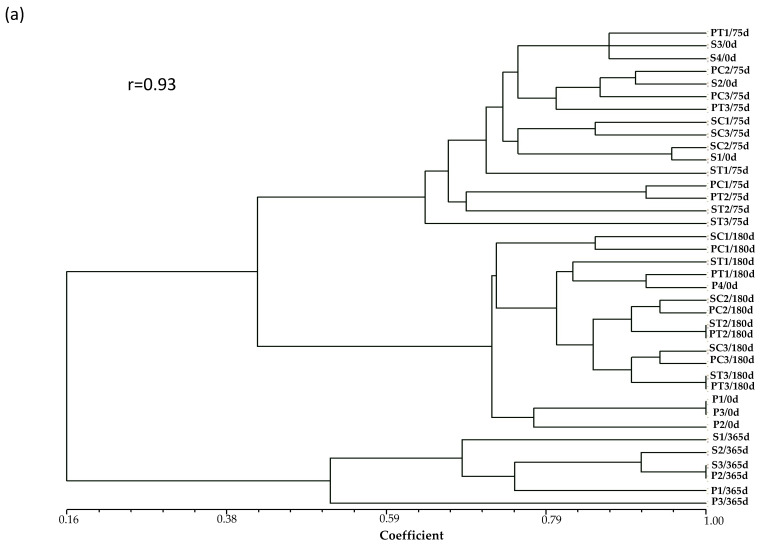

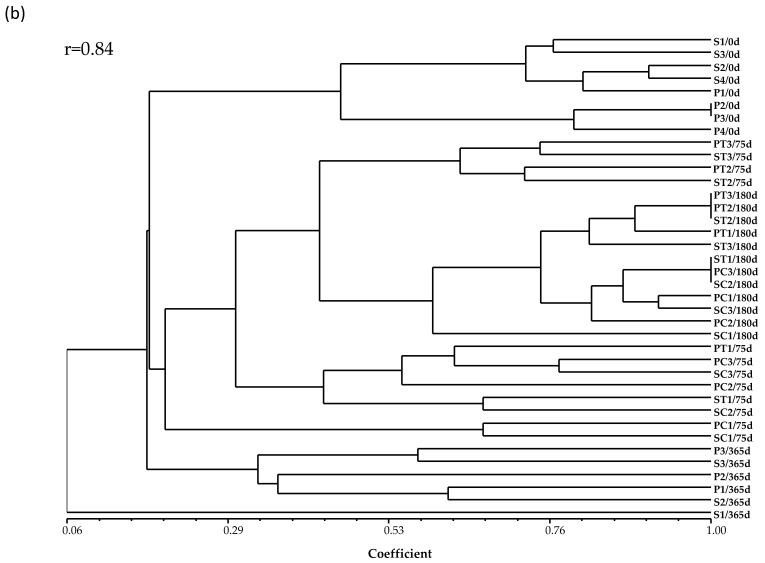
Dendrograms comparing similarity levels of the fungi community in commercial orchards in Madrid in (**a**) Villaviciosa de Odón (VO) and (**b**) La Canaleja (LC). Cluster analysis was developed using the UPGMA method based on Dice similarity coefficient genetic distances between soil fungal populations sampled at 0, 75, 180 and 365 days from transplanting to the seedbed at two depths, S (0–5 cm) and P (6–10 cm). Treatments were applied to tomato seedlings in seedbed 7 days before transplanting and consisted of dried conidia of PO212 (T) and untreated control (C). SC samples taken at surface from soil in control plants; PC samples taken at depth from soil in control plants; ST samples taken at surface from soil in dried conidia of PO212-treated plants; and PT samples taken at depth from soil in dried conidia of PO212-treated plants. Three samples (1–3) per treatment were analyzed, except for days 0 and 365, where four samples (1–4) were analyzed. The scale represents the genetic similarity (1–100%) between samples. r, represents the cophenetic correlation coefficient.

Fungal community composition varied significantly over time and with soil depth, but no clear changes could be attributed to PO212 application. In DGGE profile comparisons, samples consistently clustered by sampling date rather than by treatment (Figure 1a,b). For instance, in both VO and LC soils, the profiles from 365 days after transplanting formed a distinct cluster, clearly separated from the 0-, 75-, and 180-day profiles, regardless of PO212 treatment. UPGMA dendrograms based on Dice similarity coefficient further supported this observation. Communities from the same time point and soil depth clustered tightly together, with treated and control samples intermingled and showing high similarity (Figure 1). Likewise, neighbor-Joining cluster analysis based on Nei’s distances yielded the same pattern: no grouping by treatment was evident. Instead, the most pronounced shifts in community composition aligned with seasonal progression from planting to harvest and one year after transplanting.

### 3.2. Temporal and Spatial Dynamics vs. Treatment Effects

Table 1 shows the results of the AMOVA analyses for the fungal populations of the commercial orchards VO (a) and LC (b). These results corroborated the above observations.

The proportion of total variance in fungal community composition attributable to PO212 treatment was very low (<1%) and not statistically significant (*p* ≫ 0.0001). In contrast, sampling date explained a substantial and statistically significant portion of the variance (approximately 20–30%, *p* < 0.01), while soil depth accounted for an additional significant portion of variance (~10–15%) in both field trials. Differences between surface (0–5 cm) and subsurface (6–10 cm) communities were significant in some cases, whereas differences between PO212-treated and control samples were consistently non-significant (F_ST_ ≈ 0) (Table 1). Importantly, none of the three-way interactions (treatment × depth × time) were significant, indicating that the PO212 treatment had no hidden time-dependent or depth-dependent effects either.

**Figure 2:**
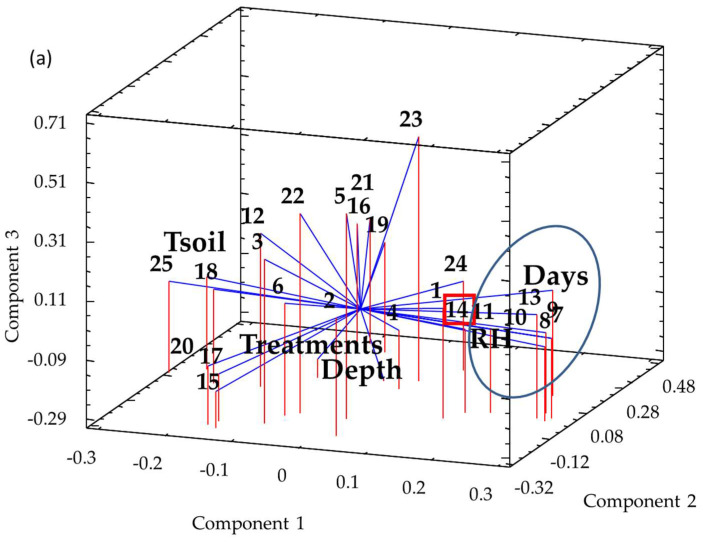

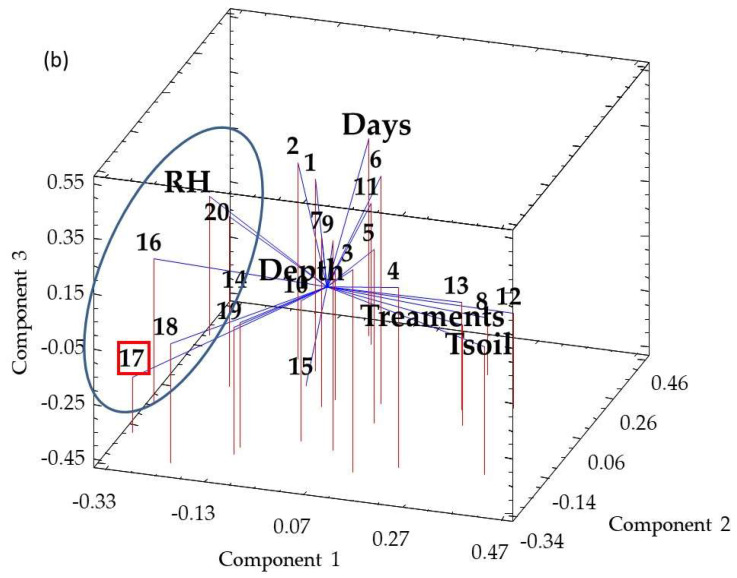
Three-dimensional representation of the principal component analysis (PCA) based on the genetic profiles of fungal populations identified in soil samples from commercial fields in Madrid (**a**) Villaviciosa de Odón (VO) and (**b**) La Canaleja (LC). Samples were collected at different time points (Days) after transplanting, at two depths, and under two Treatments (control and PO212 treated), while considering soil temperature (Tsoil) and average humidity (RH). The numbers within the figure represent the numbered and cut bands in the respective DGGE gels (Figure S1a,b). Number 14 in (**a**) and 17 in (**b**) represented the band corresponded positive control with PCR_DNA fronm PO212 culture. The values of the axes represent the percentage of the total variance explained by each dimension. The axes show the first three components of the analysis that explain 36.7%, 7.1% and 19.5% of the variation in VO and 18.6%, 18.3% and 16.3% in LC.

PCA further confirmed that the main axes of variation in fungal community composition were driven by temporal dynamics, likely reflecting seasonal environmental factors (T soil and RH). The data on temperature and RH in VO and LC are shown in Table S2a and S2b, respectively. In the PCA ordination, samples were primarily segregated along PC1 according to sampling time (with samples corresponding to 365 days separated from earlier time points), and along PC2 according to differences in soil depth (Figure 2a,b). PO212-treated samples were interspersed with controls, showing no consistent shift attributable to treatment. PC1 was strongly correlated with seasonal soil temperature trends (which declined toward 365 d), while PC2 correlated with variation in moisture and organic inputs between surface and deeper soil. The presence or absence of PO212 did not significantly load onto any principal component, reinforcing that PO212 addition did not generate a distinct community signature.

By the final sampling period (365 days), PO212 DNA was still detectable in thesoil; however, the fungal community in treated plots remained highly similar to that in the control plots (treated with sterile distilled water). Seasonal change was the primary driver of fungal community composition, affecting both treated and untreated plots equally from the crop-growing period (spring/summer) to the off-season (winter). Certain filamentous fungi, such as some saprophytic Ascomycetes, for instance, became more abundant by day 365 across all plots, likely due to the decomposition of crop residues after the growing season. At the same time, some pathogenic populations declined as their host plants were no longer present. These successional changes occurred regardless of PO212 treatment.

In summary, no persistent or significant differences in the soil fungal community could be attributed to PO212. DGGE profiles and statistical analyses show that PO212 did not disrupt the structure of the native fungal community. Any minor fluctuations in community composition following PO212 application were within the range of natural temporal variability and quickly equilibrated. This is evidenced by the similarity of the profiles of the treated and control samples at each sampling time, and by the overwhelming influence of season and depth on community composition.

### 3.3. Identification of Indigenous Fungi

Sequencing of DGGE bands provided insight into the fungal taxa that co-existed with PO212 in the soil environment. In total, 413 and 168 DGGE bands were detected across samples in the VO and LC samples, respectively (Figure S1).

A total of 56 DGGE bands were extracted and sequenced from the VO samples. Of these, 84% (47 bands) were identified, corresponding to 62% (257 bands) of the total bands in the gel (see Figure S1a and Table S3). From the LC, samples 46 DGGE bands were extracted and sequenced. Of these, 80% (37 bands) were identified, corresponding to 51% (132 bands) of the total bands in the gel (see Figure S1b and Table S4).

**Figure 3:**
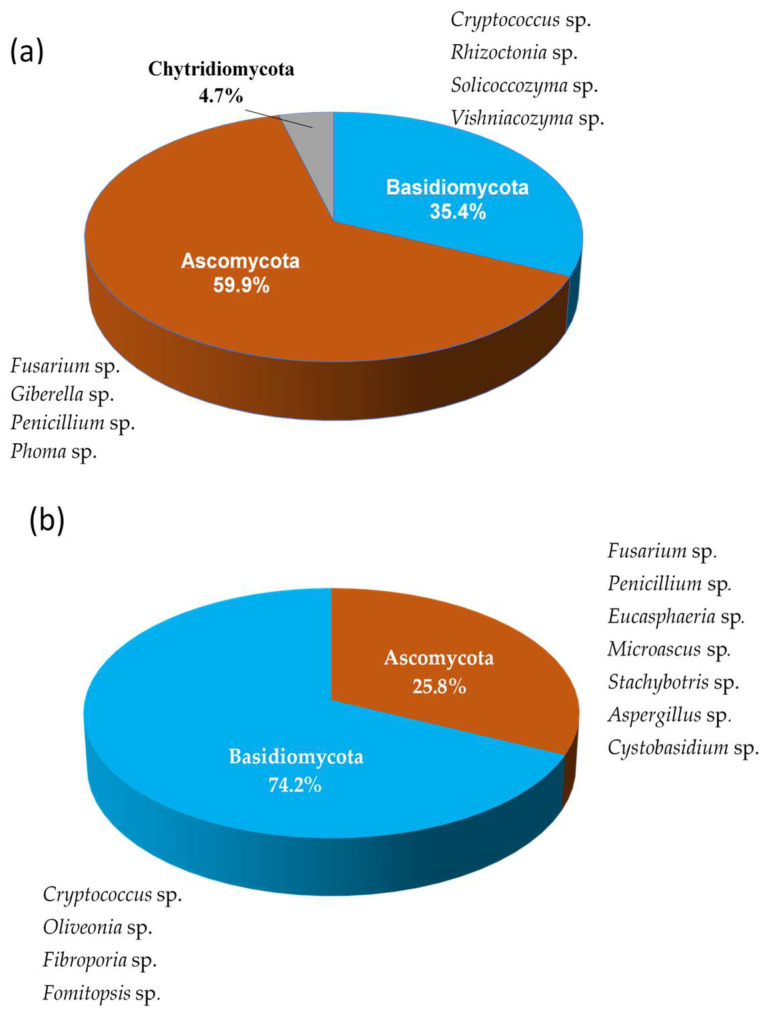
Relative abundance of main fungal phyla identified in soil samples (expressed as percentages) from commercial fields in (**a**) Villaviciosa de Odón (VO) and (**b**) La Canaleja (LC) according to the BLAST 2.17.0 of fungal ITS sequences.

Figure 3 presents the identified fungal phyla in the VO field (a) and the LC field (b). In the VO field, the phylum Ascomycota was dominant (60%), while Basidiomycota represented 35% and Chytridiomycota just 5% of the community (Figure 3a). In the LC field, meanwhile, Basidiomycota was the dominant phylum, representing 74% of the community, while Ascomycota represented only 25%. The Chytridiomycota phylum is not represented (Figure 3b).

Analysis of fungal distribution over time revealed a shift in dominant phyla (Tables S5 and S6). In the VO field, Ascomycota dominated at 0, 75, and 180 days after transplanting, representing 67.8, 63.13 and 74.4% of the total, respectively. Meanwhile, Basidiomycota was dominant at 365 days, representing 52.8% of the total. The phylum Chytridiomycota was only present in low numbers throughout the study period (Table S5). However, the phylum Basidiomycota dominated the LC field throughout the study period, reaching a value of 88% at 180 days after transplantation (see Table S5). After one year (365 days), the fungal community comprised 20% Ascomycota and 80% Basidiomycota (see Table S5). No fungi from the phylum Chytridiomycota were identified.

Fungal distribution over time in the LC field showed greater heterogeneity at the genera level compared to the VO field (Tables S6 and S7).

In the VO field, the most frequently detected genus was *Fusarium* sp., appearing in 55.8% of samples (Table S6). This was followed by *Cryptococcus* sp. (syn. *Solicoccozyma* sp.), which appeared in 28.8% of samples. Other common genera included *Gibberella* sp. (14.79%) and *Rhizoctonia* sp. (6.61%). Twelve percent of the genera included *Cercospora* sp., *Penicillium* sp., *Olpidium* sp., *Phoma* sp. and *Filobasidium* sp. At the time of transplanting (day 0), the most frequently detected genera were *Fusarium* sp. (40.5%) and *Cryptococcus* sp. (19.4%). At 75 and 180 days, *Fusarium* sp. remained the most abundant genus (38.4% and 44.8%, respectively). After one year (365 days), *Cryptococcus* sp. (41%) became the predominant genus (Tables S4 and S5). The remaining 19% corresponded to uncultured fungi and unidentified genera. At the species level, the following taxa were identified: *P. rubens*, *C. aerius* (syn. *Solicoccozyma aeria*), *C. festucosis*, *C. terreus* (syn. *Solicoccozyma terrea*), *F. oxysporum*, *F. culmorum*, *O. brassicae* and *P. herbarum*. *Penicillium rubens* was detected at 75 and 365 days after transplanting, but not at day 0.

In the LC field (Table S7), the most prominent genus was *Cryptococcus* sp. (syn. *Solicoccozyma* sp.) accounting for 63.64% of the total identified genera. This was followed by *Penicillium* sp. (13%). The remaining 27% incomprised *Oliveonia* sp., *Stachybotrys* sp., *Aspergillus* sp. and *Eucasphaeria* sp. (Table S7). *Cryptococcus* sp. (57–85%) was the predominant genus at 0, 75, 180 and 365 days after transplanting. *Penicillium* sp. followed, appearing in low but constant percentages throughout the trial (9–13%) (Table S7). At the species level, the following taxa were identified: *Cryptococcus victoriae*, *C. carnescens* and *C. chernovii*; *Oliveonia pauxilla*, *Cystobasidium slooffiae*, *Penicillium rubens* and *P*. *chrysogenum*, *Stachybotrys chartarum* and *Eucasphaeria capensis*.

Crucially, PO212 treatment did not result in the dominance of new fungal taxa, or the displacement of native taxa. All major fungal groups detected were present in both treated and untreated soils, with community composition shifting primarily over time. PO212’s ITS sequence showed high similarity to other *Penicillium*/*Talaromyces* sequences in reference databases, and its corresponding DGGE band persisted through the final sampling point, indicating that the BCA survived in the soil but did not outcompete or disrupt the native fungal community.

## 4. Discussion

These field trials indicate that introducing PO212 into the soil of a tomato field has an insignificant impact on the indigenous fungal community. Over the course of one year, the soil fungal populations in PO212-treated plots remained statistically indistinguishable from those in untreated controls, both in terms of community structure and diversity. Any minor differences observed in fungal profiles were attributable to natural seasonal dynamics rather than the biocontrol treatment itself. This finding is encouraging for the environmental risk assessment of PO212, as it suggests that this BCA can be used to manage diseases without disrupting the balance or function of the native soil microbiome.

The experiment was designed to evaluate potential impacts of PO12 on non-target soil microbial communities. The soil sampling times (0, 75, 180 and 365 days after transplanting) were chosen to capture the main phases of tomato crop development and the corresponding shifts in soil microbial activity. Sampling at 0 days provides the baseline community before crop establishment. The 75-day sampling corresponds to peak vegetative growth, when microbial-driven nutrient cycling is most active. Sampling at 180 days represents the end of the cropping cycle, when root senescence and residue incorporation can strongly influence fungal composition and the plants are removed. The final sampling at 365 days assesses longer-term dynamics and the potential persistence or recovery of the soil fungal community one year after planting. Together, these time points allow evaluation of both short-term and longer-term effects of the treatment on soil fungi.

The most significant changes in the fungal community composition were associated with the sampling date (i.e., environmental conditions) and soil depth. This is consistent with the patterns reported in other studies of soil microbial communities. Seasonal fluctuations in temperature, moisture, and plant root activity are known to drive successional changes in soil fungi. In our study, fungal profiles obtained during winter sampling (365 days) clustered separately from those collected in spring and summer (0–180 days). This reflects a seasonal shift, whereby colder temperatures and the presence of the decomposing plant residues in winter favoured certain saprophytic fungi. Similar temporal clustering patterns have been documented in previous studies [39,40], which demonstrate that soil microbial communities are highly responsive to environmental conditions and exhibit predictable seasonal trends. For example, studies in maize fields have shown that the composition of the fungal community varies with soil temperature and moisture regimes, independently of experimental treatments [41]. Likewise, it was reported that annual changes in soil physicochemical properties, such as nutrient availability and organic matter inputs, can influence microbial biomass and community structure, further emphasizing the role of seasonal cycles. In our trials, differences between surface and subsurface fungal communities were also apparent, likely due to gradients in organic carbon, oxygen availability, and moisture. These findings highlight the importance of accounting for natural spatio-temporal variability when assessing the ecological impact of BCAs. Establishing such a baseline is essential for distinguishing treatment effects from background environmental variation.

Crucially, PO212 inoculation did not cause shifts that went beyond the range of natural variability. The AMOVA and clustering analyses confirmed that the variation attributable to PO212 treatment was effectively zero, whereas temporal and depth-related factors had significant effects. In practical terms, the fungal community in a PO212-treated soil at a given time point was more similar to that in an untreated soil from the same time point than to its own community at a different time. This finding aligns with previous research on other BCAs. For instance, Savazzini et al. (2009) [12] examined the effects of applying *T. atroviride* strain SC1 to soil in a vineyard. They found that, over the long term, soil depth had a greater influence on composition of the fungal community than the *Trichoderma* treatment itself. Although *T. atroviride* SC1 is known to produce various antibiotics and lytic enzymes that can initially disturb nearby microbes, subsequent work [42] demonstrated that it did not significantly alter fungal community diversity over an extended period. Our results with PO212 reflect this behavior: any initial disturbance to the fungal community was transient, with no long-term shifts observed. A more recent study by Leal et al. (2024) [43] on *T. atroviride* (SC1 strain) reported a modest, short-term decrease in fungal species richness in certain soils following its application. However, even in that case, the changes were context-dependent and often temporary. Environmental variables such as soil type were found to influence SC1’s impact on native fungal populations. This finding is consistent with our observation that inherent site differences (VO vs. LC) had a greater influence on community composition than the BCA treatment.

In this regard, the higher number of DGGE bands in VO compared with LC likely reflects site-specific differences in soil properties and management history, which are known to influence fungal richness and DGGE band complexity. The substantially higher number of DGGE bands observed in VO (413) compared with LC (168) likely results from underlying differences in soil properties and agronomic history between both sites. Soil parameters such as organic matter, texture, nutrient availability, moisture regime, pH and previous crop management are known to modulate fungal diversity. VO soils are usually less disturbed, which favors a more complex fungal community. In contrast, LC soils may undergo more intensive management, which reduces fungal richness. These environmental and management-driven differences probably account for the contrasting band number

In mesocosm experiments conducted by Postma’s [5], the introduction of *T. atroviride* strain I-1237 caused a temporary increase in culturable fungal counts. However, this effect was quickly overshadowed by fluctuations driven by sampling time and environmental variations such as changes in soil water content and aeration, which had a greater influence on microbial populations than the BCA itself. Our field data and other studies by Larena et al. (2014) [20], support this conclusion. The influence of PO212was minimal compared to the effects of seasonal drying and rewetting cycles.

Similarly, studies by Cordier et al. (2009) and Brocket et al. (2012) [44,45] also highlighted soil moisture and aeration as key drivers of microbial community composition. In our PCA, soil moisture emerged as a significant factor influencing the distribution of certain fungal groups in all samples, including those associated with the presence of PO212. Notably, the LC field had lower soil moisture and a different fungal community to the VO soil, suggesting that seasonal climate differences could influence BCA integration. Nevertheless, neither environment exhibited any harmful effect of PO212; there was no evidence of the competitive displacement of beneficial fungi or the proliferation of opportunistic organisms that could disrupt ecosystem functions.

From a biocontrol efficacy perspective, it is encouraging that PO212 persisted in the soil (its DNA was detectable for up to one year) and presumably continued to suppress the pathogen *F. oxysporum* [30]. Notably, when PO212 is applied, the number of fungal colony-forming unit (CFUs) in the rhizosphere initially increases, but then returns to background levels (3 × 10^2^ CFU/g dry soil) within three to five months after application as described by Vázquez et al. [15]. This pattern is consistent with results reported for other BCAs like *Trichoderma* spp. and *Beauveria bassiana* [8], demonstrating that BCA can coexist with native soil fungi without displacing them. In fact, several native fungi considered beneficial (e.g., saprophytes involved in nutrient cycling) remained abundant throughout the study. This suggests that PO212 achieves a desirable balance: it is competitive enough to survive and protect plants, but not so aggressive as to dominate or disrupt the existing microbial community.

This ecological compatibility is likely related to PO212’s mode of action, which mainly involves inducing resistance in plants. It has previously been demonstrated that PO212 induces resistance in tomato plants, possibly via its xylanolytic enzyme system [46]. This mechanism was not demonstrated in the present study and therefore cannot be confirmed from our result. This specific biological control strategy may help to explain the minimal collateral impact observed in our soil microbial assessments. The effectiveness of microbial biocontrol agents (BCAs) can vary with environmental conditions, which has contributed to slower adoption compared with conventional fungicides. While regulatory frameworks—originally designed for chemical pesticides—also play a role, the key factor for wider acceptance is demonstrating that a BCA poses minimal risk to soil health and non-target organisms. Our results provide evidence that PO212 does not negatively affect these components of the ecosystem, supporting its environmental safety. As regulatory frameworks increasingly classify BCAs as “low-risk” products with streamlined requirements, such ecological data can help facilitate the approval of PO212 as a registered biopesticide.

This study, which was conducted in two locations in Madrid with distinct soil characteristics and management histories, demonstrates that the application of PO212 did not cause major disruptions to soil fungal populations under both tested conditions. The dose and frequency of PO212 application used in this study are those that have previously been shown to be effective in reducing tomato wilt [18,19,20,21].

In summary, the application rate and method used in this study reflect real field-use conditions for PO212, supporting the relevance of our findings and demonstrating its effectiveness and safety under practical agricultural application. The application of PO212 in tomato field soils did not significantly disrupt the native soil fungal community. Observed variations in fungal populations were attributed to natural seasonal changes rather than to the presence of the biocontrol fungus. This finding is crucial for environmental risk assessment, as it indicates that PO212 is unlikely to interfere with essential soil ecological processes or negatively affect beneficial microbes. These results support the broader view that BCA when properly selected and applied; can be compatible with soil ecosystem integrity [45].

## 5. Conclusions

This study represents a first step toward understanding the structure and variability of soil fungal communities following the introduction of the BCA PO212. Our findings highlight the importance of evaluating microbial BCA not only for their efficacy against plant pathogens, but also for their ecological compatibility with native soil microbiota and their potential effects on ecosystem services. Using the culture-independent technique PCR-DGGE, we were able to monitor microbial dynamics over time and generate comparative profiles of the dominant fungal taxa present in different soils under various treatment conditions. The results showed that PO212 successfully established itself as a member of the soil microbiota without displacing or outcompeting the indigenous fungi. This indicates that there was minimal disturbance to the existing fungal communities. Our findings strongly support that PO212 is a safe and eco-friendly tool for managing Fusarium wilt and potentially other soil-borne diseases. Widespread use of such BCA, in combination with soil health monitoring, could help reduce reliance on chemical pesticides while preserving the essential functions of soil microbiomes.

## Figures and Tables

**Table 1:** Analysis of molecular variance (AMOVA) of fungal populations in soil samples from commercial fields in Madrid Villaviciosa de Odón (VO) and La Canaleja (LC).


**Variation Source**	**d.f.**	**Variance Components**	**% Total Variation**	***F*** **_st_**	***p***
All distances: two groups					
Between depths	1	0	0		
Within depths	36	4.49	100	−0.004	0.447
All treatments: two groups					
Between treatments	1	0.16	4		
Within treatments	36	4.41	96	0.036	0.124
All sampling dates: four groups					
Between sampling dates	3	2.76	53		
Within sampling dates	34	2.41	47	0.534	0.0001 *

**Variation Source**	**d.f.**	**Variance Components**	**% Total Variation**	***F*** **_st_**	***p***
All distances: two groups					
Between depths	1	0	0	-	
Within depths	36	3.5	100	0.034	0.987
All treatments: two groups					
Between treatments	1	0.38	11		
Within treatments	36	3.27	89	0.105	0.003
All sampling dates: four groups					
Between sampling dates	3	1.55	41		
Within sampling dates	34	2.28	59	0.406	0.0001 *

d.f. degrees of freedom; * Significant for a value of *p* based on 10,000 permutations.

## Data Availability

The original contributions presented in this study are included in the article/Supplementary Materials. Further inquiries can be directed to the corresponding author.

## Supplementary Materials

The following supporting information can be downloaded at: https://www.mdpi.com/article/10.3390/jof11120852/s1, Figure S1: Banding profiles obtained after DGGE of soil samples from (a) Villaviciosa de Odón (VO) and (b) La Canaleja (LC), collected at 0, 75, 180, and 365 days after transplanting. Soil was sampled from plants treated with dried conidia of PO212 (P) and untreated controls (C), at two depth levels: surface (S: 0–5 cm) and deepth (P: 6–10 cm). Each lane corresponds to one soil sample. PO is P. rubens strain 212 (PO212) profile; M: reference marker mix (50 bp ladder, Invitrogen). Arrows with a number indicate the position of bands that were cleaved from the gels for subsequent sequencing. SC are soil samples taken at surface from the control plants; PC are soil samples taken at depth from the control plants; SP are soil samples taken at surface from the with dried conidia of PO212-treated plants; and PP are those taken at depth from the with dried conidia of PO212 treated plants. The numbers for each sample type indicate that three rrplicates (1,2,3) were taken (n = 3); Table S1: The physical and chemical properties of the natural soils of two commercial sites, Villaviciosa de Odón (VO) and La Canaleja (LC) both of which were located near Madrid, Spain, that were used in the two field trials; Table S2: Temperatures and humidity recorded at the (a) Villaviciosa de Odón and (b) La Canaleja during the trial period; Table S3: Phylogenetic affiliation of fungal ITS sequences from commercial fields in Villaviciosa de Odón (VO), Madrid, after DGGE at the 0, 75, 180 and 365 days after transplanting. Only bands with ≥92% similarity to the reference sequences in the databases are shown. Distribution of fungal phyla over time; Table S4: Phylogenetic affiliation of fungal ITS sequences from commercial fields in La Canaleja (LC), Madrid, after DGGE at the 0, 75, 180 and 365 days after transplanting. Only bands with ≥92% similarity to the reference sequences in the databases are shown. Distribution of fungal phyla over time; Table S5: Percentage of main phyla of fungi identified from commercial fields in Villaviciosa de Odón (VO), and La Canaleja (LC) Madrid, after DGGE at the 0, 75, 180 and 365 days after transplanting; Table S6: Percentage of main genera of fungi identified from commercial fields in Villaviciosa de Odón (VO) after DGGE at the 0, 75, 180 and 365 days after transplanting; Table S7: Percentage of main genera of fungi identified from commercial fields in La Canaleja (LC), Madrid, after DGGE at the 0, 75, 180 and 365 days after transplanting.

## Abbreviations

The following abbreviations are used in this manuscript:

BCA	Biocontrol agent
AMOVA	Analysis of molecular variance
AM	arbuscular mycorrhizal
C	Untreated control
CSPO	Dry powder inocula
LC	Field trial experiment-La canaleja
OTU	Operational taxonomic unit
P	6–10 cm soil depth
PCA	Principal component analysis
PCR-DGGE	Polymerase chain reaction-denaturing gradient gel electrophoresis
PO212	Penicillium rubens strain PO212
S	0–5 cm soil depth
UPGMA	UPGMA (unweighted pair group method with arithmetic mean
VO	Field trial experiment-Villaviciosa de Odón
