# Supplementary material for: Environmental Compatibility of Penicillium rubens Strain 212: Impact on Indigenous Soil Fungal Community Dynamics
Source: J Fungi (Basel). 2025 Nov 29;11(12):852. doi: 10.3390/jof11120852 (PMC12733579; doi:10.3390/jof11120852)
Supplement: Supplementary file 1 [file jof-11-00852-s001.zip › jof-3956043-supplementary.pdf]

Supplementary Material

Environmental compatibility of *Penicillium rubens* strain 212: impact on indigenous soil fungal community dynamics

Belén Guijarro [1], Gema Vázquez [1], Antonieta De Cal[1], Paloma Melgarejo[1], Nuria Gaju[2], Maira Martínez-Alonso[2] and Inmaculada Larena [1*]

1 Grupo de Hongos Fitopatógenos, Departamento de Protección Vegetal, Centro Nacional INIA-CSIC, Madrid, Spain

2 Departament de Genètica i Mcrobiologia. *Universitat Autònoma de Barcelona, 08193 Bellaterra, Barcelona, Spain*

* Correspondence: ilarena@inia.csic.es

Table S1.

The physical and chemical properties of the natural soils of two commercial sites, Villaviciosa de Odón (VO) and La Canaleja (LC) both of which were located near Madrid, Spain, that were used in the two field trials.

	VO[a]	LC[b]
Humidity (%)	3.19	11
pH (1:2.5)	7.6	8.5
EC (1:5) μS/cm	169	180
OM content (%)	0.91	1.11
Texture	Sandy loam soil	Loamy sand soil
% Sand (2-0.02 mm)	84.8	63.4
% Silt (0.02-0.002 mm)	8.7	22.3
% Clay (< 2 μm)	6.5	11
Available *P* (mg/kg)	46.9	39.0
exchangeable K^+ (mmol/kg)	2.9	17.94
exchangeable Mg^+ (mmol/kg)	6.2	13.3
exchangeable Na^+ (mmol/kg)	2.6	3.7
Total nitrogen (%)	0.09	0.14
$CaCO_3$ (%)	2.2	10.9

EC- Electrical Conductivity; OM- Organic Matter. For pH, the numbers in parentheses refer to 1:2.5 (w/v) aqueous soil suspensions. For EC, the numbers in parentheses refer to a 1:5 (w/v) aqueous soil suspension. For texture, the numbers in parentheses refer to particle size (mineral fractions in soil). [a]VO, soil from a commercial orchard in Villaviciosa de Odón, southern Madrid, Spain (Coordinates: 40°21′17″ N, 3°53′55″ W),. [b]LC, soil from the commercial orchard in La Canaleja farm , Alcalá de Henares, eastern Madrid, Spain (Coordinates: 40°28′53″ N, 3°21′50″ W)

(a)

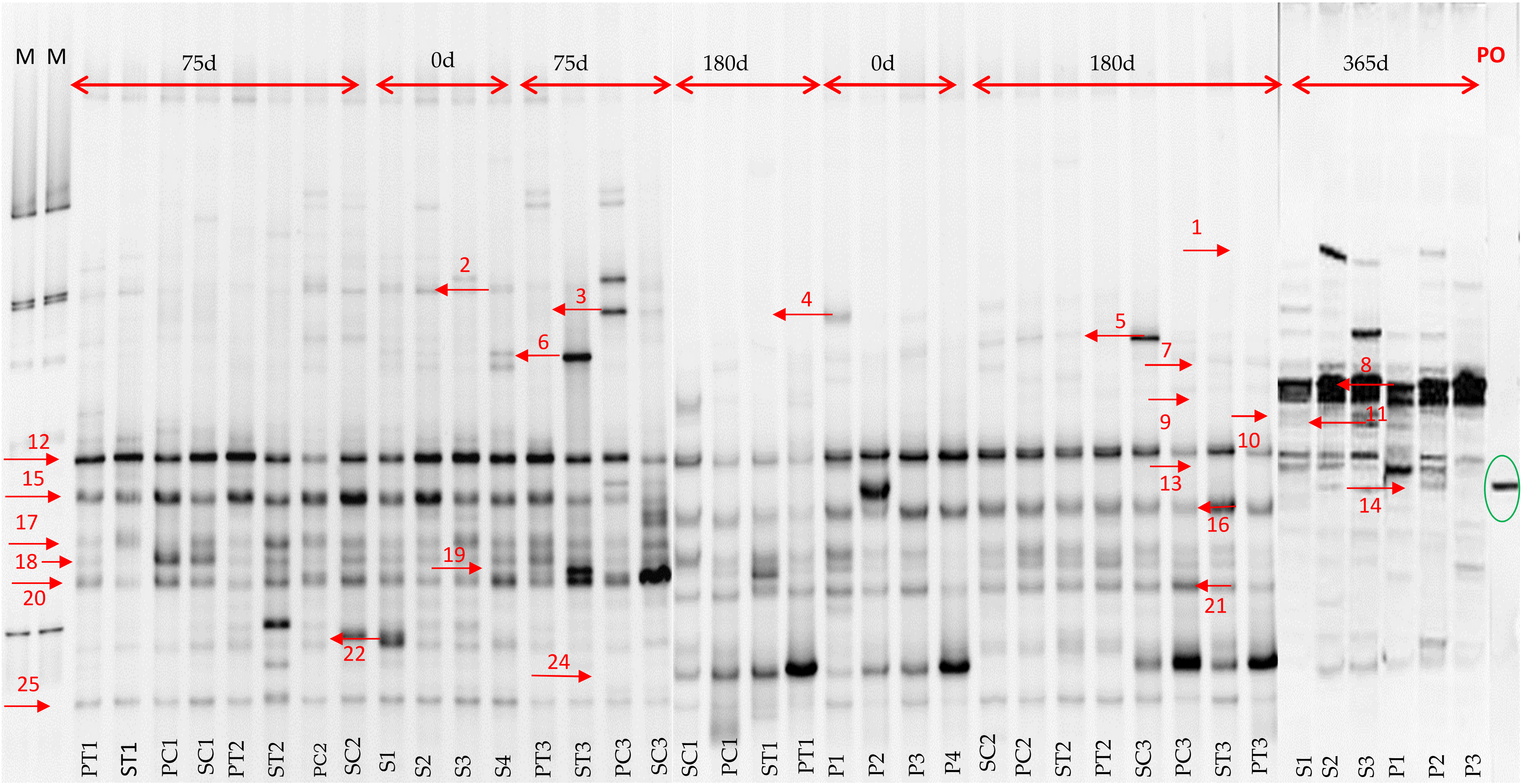
M
M
75d
0d
75d
180d
0d
180d
365d
PO
1
2
3
4
5
6
7
8
9
10
11
12
13
14
15
16
17
18
19
20
21
22
24
25
PT1
ST1
PC1
SC1
PT2
ST2
PC2
SC2
S1
S2
S3
S4
PT3
ST3
PC3
SC3
SC1
PC1
ST1
PT1
P1
P2
P3
P4
SC2
PC2
ST2
PT2
SC3
PC3
ST3
PT3
S1
S2
S3
P1
P2
P3

(b)

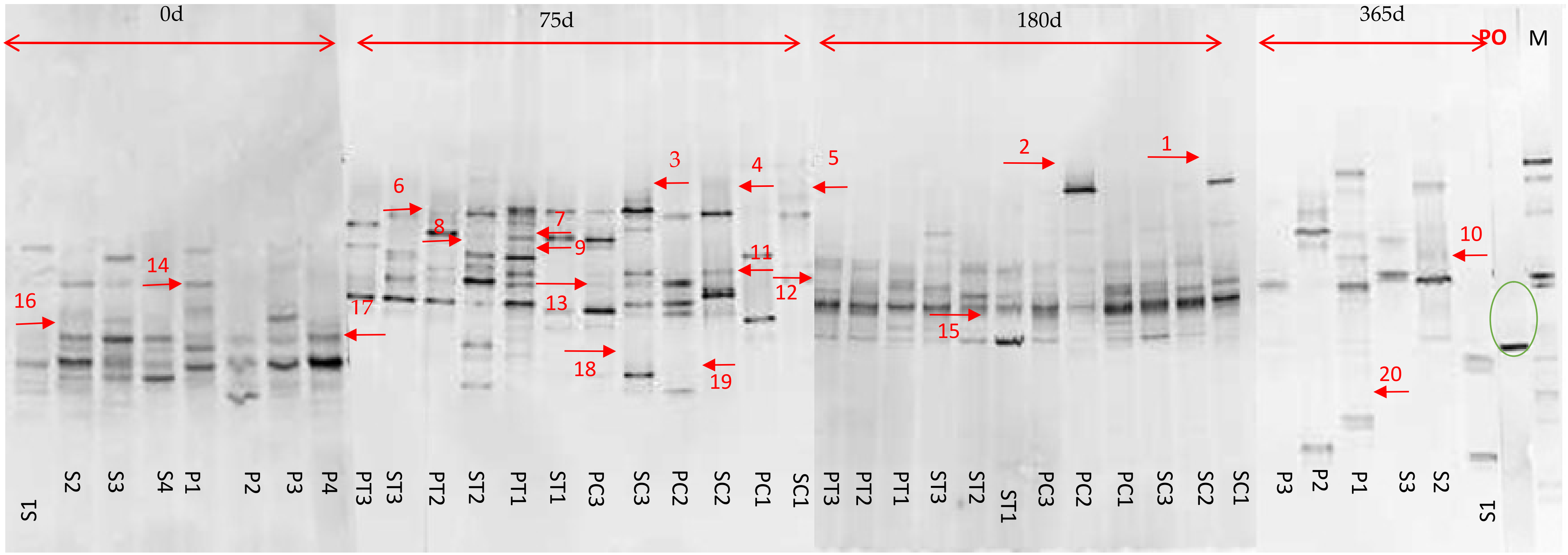

0d
75d
180d
365d
PO
M
S1
S2
S3
S4
P1
P2
P3
P4
PT3
ST3
PT2
ST2
PT1
ST1
PC3
SC3
PC2
SC2
PC1
SC1
PT3
PT2
PT1
ST3
ST2
ST1
PC3
PC2
PC1
SC3
SC2
SC1
P3
P2
P1
S3
S2
S1
1
2
3
4
5
6
7
8
9
10
11
12
13
14
15
16
17
18
19
20

Figure S1. Banding profiles obtained after DGGE of soil samples from (a) Villaviciosa de Odón (VO) and (b) La Canaleja (LC), collected at 0, 75, 180, and 365 days after transplanting. Soil was sampled from plants treated with dried conidia of PO212 (P) and untreated controls (C), at two depth levels: surface (S: 0–5 cm) and deepth (P: 6–10 cm). Each lane corresponds to one soil sample. PO is *P. rubens* strain 212 (PO212) profile; M: reference marker mix (50 bp ladder, Invitrogen). Arrows with a number indicate the position of bands that were cleaved from the gels for subsequent sequencing. SC are soil samples taken at surface from the control plants; PC are soil samples taken at depth from the control plants; SP are soil samples taken at surface from the with dried conidia of PO212-treated plants; and PP are those taken at depth from the with dried conidia of PO212 treated plants. The numbers for each sample type indicate that three rrplicates (1,2,3) were taken (n = 3).

Table S2. Temperatures and humidity recorded at the (a) Villaviciosa de Odón and (b) La Canaleja during the trial period

(a)

Days after trasplanting	Air temperature (°C)	Soil temperature (°C)	Mean humidity (%)
0 (4th My)	13.4	14.8	60.4
75	23.46	27.0	47.4
180	17.39	19.49	58.7
365	9.7	10.67	72.0

(b)

Days after trasplanting	Air temperature (°C)	Soil temperature (°C)	Mean humidity (%)
0 (25th May)	16.45	18.36	49.27
75	22.62	25.95	49.27
180	23.10	26.57	47.63
365	8.85	9.74	70.53

Table S3. Phylogenetic affiliation of fungal ITS sequences from commercial fields in Villaviciosa de Odón (VO), Madrid, after DGGE at the 0, 75, 180 and 365 days after transplanting. Only bands with ≥92% similarity to the reference sequences in the databases are shown. Distribution of fungal phyla over time.

Band Position	Total number of bands in gel	Number of Bands Cut and sequenced	Closest BLAST Match	Homology	Phylum	Band Presence/Absence			
						0 days	75 days	180 days	365 days
1	6	4	*Olpidium brassicae*	95%	Chytridiomycota	+	-	-	+
2	14	1	*Solicoccozyma terrea* (Syn. *Cryptococcus terreus*)	96%	Basidiomycota	+	+	-	+
3	6	1	*Olpidium brassicae*	94%	Chytridiomycota	+	+	-	-
5	14	3	*Solicoccozyma terrea* (Syn. *Cryptococcus terreus*)	96%	Basidiomycota	+	+	+	+
6	4	1	*Phoma herbarum*	93%	Ascomycota	+	+	-	-
7	6	1	*Solicoccozyma terrea* (Syn. *Cryptococcus terreus*)	92%	Basidiomycota	-	-	-	+
8	6	2	*Solicoccozyma terrea* (Syn. *Cryptococcus terreus*)	98-99%	Basidiomycota	-	-	-	+
10	7	2	*Solicoccozyma area* (Syn. *Cryptococcus aerius*)	96-98%	Basidiomycota	-	-	+	+
11	6	1	*Solicoccozyma terrea* (Syn. *Cryptococcus terreus*)	96%	Basidiomycota	-	+	+	+
12	38	6	*Gibberella* sp	93%	Ascomycota	+	+	+	+
13	5	3	*Cryptococcus festucosis*	97%	Basidiomycota	-	-	-	+
**14**	**7**	**2**	***Penicillium rubens***	**99%**	Ascomycota	+	+	-	+
15	16	2	*Cryptococcus* sp.	97-99%	Basidiomycota	+	+	-	-
17	15	2	*Fusarium oxysporum*	99%	Ascomycota	+	+	-	-
18	27	3	*Fusarium culmorum*	96%	Ascomycota	+	+	+	-
19	16	4	*Fusarium sp.*	93%	Ascomycota	+	+	+	+
20	18	5	*Fusarium sp.*	97-100%	Ascomycota	+	+	+	-
21	16	1	*Fusarium* sp.	99%	Ascomycota	+	-	+	-
23	13	1	*Cercospora* sp.	93%	Ascomycota	+	-	+	
24	17	2	*Rhizoctonia* sp.	99%	Basidiomycota	+	+	+	+
	240	45							

+ band present; - band absent- Positions (4, 9, 16, 22 and 25) that do not appear on the list are not shown because they could not be identified.

Table S4. Phylogenetic affiliation of fungal ITS sequences from commercial fields in La Canaleja (LC), Madrid, after DGGE at the 0, 75, 180 and 365 days after transplanting. Only bands with ≥92% similarity to the reference sequences in the databases are shown. Distribution of fungal phyla over time.

Band Position	Total number of bands in gel	Number of Bands Cut and sequenced	Closest BLAST Match	Homology	Phylum	Band Presence/Absence			
						0 days	75 days	180 days	365 days
1	2	2	*Cystobasidium slooffiae*	96%	Ascomycota	-	-	+	+
2	3	1	*Fomitopsis* sp.	100%	Basidiomycota	-	-	+	+
5	4	1	*Fibroporia radiculosa*	99%	Basidiomycota	-	+	-	+
6	8	3	*Solicoccozyma aeria* (Syn. *Cryptococcus aerius)*	99%	Basidiomycota	-	+	+	+
7	7	3	*Oliveonia pauxilla*	95%	Basidiomycota	+	+	-	+
8	15	1	*Cryptococcus chernovii*	92%	Basidiomycota	-	+	+	-
9	4	3	*Vishniacozyma carnescens* (Syn. *Cryptococcus carnecens)*	99%	Basidiomycota	-	+	-	+
10	21	5	*Cryptococcus* sp	99%	Basidiomycota	+	+	+	+
11	9	1	*Penicillium chrysogenum*	98%	Ascomycota	-	+	+	+
12	19	4	*Cryptococcus victoriae*	99%	Basidiomycota	-	+	+	-
15	17	4	*Cryptococcus sp.*	98%	Basidiomycota	+	-	+	-
16	5	2	*Stachybotrys chartarum*	100%	Ascomycota	+	+	-	-
17	8	3	*Penicillium rubens*	98%	Ascomycota	+	-	-	-
18	5	3	*Aspergillus* sp.	98%	Ascomycota	+	+	-	-
19	4	2	*Eucasphaeria capensis*	98%	Ascomycota	+	+	-	-
20	1	1	*Microascus* sp.	98%	Ascomycota	-	-	-	+
	132	37							

+ band present; - band absent- Positions (3, 4, 13 and 14,) that do not appear on the list are not shown because they could not be identified.

Table S5. Percentage of main phyla of fungi identified from commercial fields in Villaviciosa de Odón (VO), and La Canaleja (LC) Madrid, after DGGE at the 0, 75, 180 and 365 days after transplanting

	Villaviciosa de Odón (VO)					La Canaleja (LC)				
Phylum	TOTAL	**0d**	**75d**	**180d**	**365d**	TOTAL	**0d**	**75d**	**180d**	**365d**
Chytridiomycota	4.70	5.29	3.03	-	4.22	-	-	-	-	-
Ascomycota	59.90	67.84	63.13	74.42	42.96	25.76	32.84	22.77	11.70	20.34
Basidiomycota	35.40	26.87	33.84	25.58	52.82	74.24	67.16	77.23	88.30	79.66
	100	100	100	100	100	100	100	100	100	100

Table S6. Percentage of main genera of fungi identified from commercial fields in Villaviciosa de Odón (VO) after DGGE at the 0, 75, 180 and 365 days after transplanting

		Main fungal genera (%)				
**Phylum**	**Fungal Genera**	**TOTAL**	**0d**	**75d**	**180d**	**365d**
Chytridiomycota	*Olpidium* sp.	4.67	5.29	3.03	0	4.22
Basidiomycota	*Cryptococcus* sp.	28.79	19.38	25.25	15.7	40.84
	Rhizoctonia sp.	6.61	7.49	8.59	9.88	11.97
	Gibberella sp.	14.79	16.74	19.19	22.09	26.76
Ascomycota	*Penicillium* sp.	2.72	3.08	3.53	0	4.93
	Phoma sp.	1.56	1.76	2.02	0	0
	Fusarium sp.	35.8	40.53	38.38	44.77	11.27
	Cercospora sp.	5.06	5.73	0	7.56	0
		100	100	100	100	100

Table S7. Percentage of main genera of fungi identified from commercial fields in La Canaleja (LC) Madrid, after DGGE at the 0, 75, 180 and 365 days after transplanting

		Main fungal genera (%)				
**Phylum**	**Fungal Genera**	**TOTAL**	**0d**	**75d**	**180d**	**365d**
Basidiomycota	*Fomitopsis* sp.	2.27	0	0	3.19	4.41
	Cryptococcus sp.	63.64	56.72	66.34	85.11	61.76
	Fibroporia sp.	3.03	0	3.96	0	5.88
	Oliveonia sp.	5.3	10.45	6.93	0	10.29
Ascomycota	*Penicillium* sp.	12.88	11.94	8.91	9.57	13.23
	Stachybotrys sp.	3.79	7.46	4.95	0	0
	Aspergillus sp.	3.79	7.46	4.95	0	0
	Eucasphaeria sp.	3.03	5.97	3.96	0	0
	Microascus sp.	0.76	0	0	0	1.47

	Cystobasidium sp.	1.51	0	0	2.13	2.94
		100	100	100	100	100
